# Mental Disorders and Emotional Competence Among Chinese Adolescents Before and During COVID-19 Pandemic: A Longitudinal Mediation Model

**DOI:** 10.3389/fpubh.2021.767004

**Published:** 2021-12-10

**Authors:** Wei Shi, Guangzhe Frank Yuan, Brian J. Hall, Xiaoli Liu, Ya Su, Li Zhao, Peng Jia

**Affiliations:** ^1^Institute for Disaster Management and Reconstruction (IDMR), Sichuan University, Chengdu, China; ^2^Department of Psychology, Faculty of Social Sciences, University of Macau, Macau SAR, China; ^3^Global and Community Mental Health Research Group, New York University, Shanghai, China; ^4^Department of Health, Behavior and Society, Johns Hopkins Bloomberg School of Public Health, Baltimore, MD, United States; ^5^Office of Humanities and Social Sciences Administration, Sichuan University, Chengdu, China; ^6^Department of Sociology, Dickinson College, Carlisle, PA, United States; ^7^Department of Health Policy and Management, West China School of Public Health and West China Fourth Hospital, Sichuan University, Chengdu, China; ^8^Health Emergency Management Research Center. China-PUMC C.C. Chen Institute of Health, Sichuan University, Chengdu, China; ^9^School of Resources and Environmental Science, Wuhan University, Wuhan, China; ^10^International Institute of Spatial Lifecourse Epidemiology (ISLE), Wuhan University, Wuhan, China

**Keywords:** anxiety, depression, emotional competence, longitudinal mediation model, COVID-19 exposure

## Abstract

**Background:** The outbreak of the COVID-19 pandemic has had a profound influence on the mental health and well-being of individuals across the globe. Emotional competence, defined as one's ability to recognize, understand, and manage their emotions, has been found linked with mental health problems (e.g., depression and anxiety) in previous studies. However, there is limited knowledge about the direction of the association between these factors among populations exposed to COVID-19. This study examined the possible mediation relationships between depression, anxiety, emotional competence, and COVID-19 exposure among Chinese adolescents.

**Methods:** Responses from 7,958 Chinese adolescents who had previously taken part in a two-wave study before (December 23, 2019–January 13, 2020) and during COVID-19 (June 16, 2020–July 8, 2020) were analyzed (51.67% males, mean age = 11.74, SD = 2.15). Structural equation modeling with three covariates (i.e., age, gender, and ethnicity) was used to test the longitudinal mediation relationships between COVID-19 exposure and depression, anxiety via emotional competence.

**Results:** Results indicated that the prevalence of depression (38.67 to 36.74%) and anxiety (13.02 to 12.77%) decreased from Time 1 to Time 2. The T2 emotional competence significantly mediated the relationship between T2 COVID-19 exposure and T2 anxiety (indirect effect [95% CI] = 0.011 [0.004–0.019], *p* < 0.05). T2 emotional competence also significantly mediated the relationship between T2 COVID-19 exposure and T2 depression (indirect effect [95% CI] = 0.013 [0.005–0.022], *p* < 0.05). The results indicated that T2 emotional competence had a significant and negative influence on T2 anxiety (β = −0.266, SE = 0.005, *p* < 0.001), and T2 depression (β = −0.326, SE = 0.029, *p* < 0.001).

**Conclusions:** This longitudinal research study demonstrated the crucial role of emotional competence in influencing the severity of long-term mental health problems, and suggested that emotional competence interventions can be conducted to improve mental well-being among Chinese adolescents exposed to COVID-19.

## Introduction

Adolescence is a vulnerable dangerous period during which mental disorders (e.g., depression and anxiety) can easily present themselves, increasing the risk of life-long mental illnesses (1). According to a report by the World Health Organization (2), 10 to 20% of adolescents suffer from mental problems worldwide, and most are underdiagnosed and undertreated (3). Being the fourth and sixth major causes of mental illness and disability among adolescents, depression and anxiety, respectively, are considered to be highly prevalent (2). For example, a meta-analysis of 17,894 subjects found that the prevalence of depression and anxiety was 17.96 and 13.99%, respectively, in Chinese adolescents (4). In the USA, anxiety is the most common mental disorder, with a prevalence of 31.9% among 10,123 adolescents aged 13–18 years (5). Additionally, an Australian research with a sample of 1,299 adolescents identified that the prevalence of depression and anxiety was 14.2 and 13.2%, respectively (6). Most previous research has applied a cross-sectional design to explore mental health and its correlates among adolescents, but this approach lacks the long-term tracking of mental health status among the target population (7, 8). Therefore, it is crucial to explore the potential mechanisms associated with long-term mental disorders and related factors among adolescents.

Many prior studies have shown that public emergencies have a profound effect on mental health (9–12). In the UK, a national longitudinal cohort study with a sample of 53,351 participants indicated that the prevalence of mental distress increased from 18.9% before COVID-19 to 27.3% during COVID-19 pandemic (13). In Switzerland, a study found that levels of anxiety and depressive symptoms significantly worsened among undergraduate students after COVID-19, compared with those before COVID-19 (14). However, research in the Netherlands showed that mental health variables remained approximately the same before and during COVID-19 among 141 adolescent students (15). Another longitudinal study among 203 Chinese students reported a significant decrease in anxiety and depression during COVID-19 lockdown (16). Furthermore, an American study, which included 322 young adolescents, found that for participants with a good mental health (i.e., fewer emotional problems before COVID-19), psychological symptoms significantly decreased during COVID-19 pandemic (17). Existing literature on mental health developing trend and related variables remained debatable before and during COVID-19 pandemic. Hence, it is necessary to further track long-term mental health status, and explore the influence of exposure to public emergencies on the mental health of Chinese adolescents, before and during COVID-19.

Emotional competence (EC) is broadly defined as an individual's ability to recognize, understand, and manage their emotions (18). In recent years, more research has underscored the vital role of EC in psychopathology, such as depression and anxiety (18–21). For example, previous meta-analyses have shown that individuals with higher EC are associated with lower levels of psychological distress, including depression and anxiety (20, 22–24). A previous study highlighted that a higher level of EC was associated with greater well-being and a lower risk of developing mental disorders (25). Moreover, an 18-h EC intervention experiment showed that improvement in EC promoted positive changes in psychological well-being (26). Most previous studies on EC applied a cross-sectional design and there has been a lack of studies examining the EC-mediated relationship between depression and anxiety based on longitudinal data. Thus, it is necessary to further explore the directional association between EC, depression, and anxiety, using a longitudinal mediation model.

Several previous theories and models have shown the potential influence mechanism of EC for mental disorders. First, according to the transdiagnostic emotion dysregulation model of mood and anxiety disorders, a triggering event could connect with the present diathesis and result in a negative or positive influence. The final psychological impact could depend on an individual's emotional style and recognition (27). Mood disorders result from emotional dysregulation of negative influences and are associated with an absence of positive affect (28). Second, the transdiagnostic models of psychopathology explain the mechanisms by which transdiagnostic risk factors result in multiple mental disorders (29). The model suggested that biological factors giving rise to potentially maladaptive emotional and cognitive trends could directly result in psychological symptoms, such as depression and anxiety (30). Finally, the ABC theory of emotion proposed by Ellis (31) holds that emotions can be directly determined by the individual evaluation and cognition processes of the triggering event. Emotional evaluation and recognition are greatly influenced by EC, which can lead to various emotional reactions and changes (20). Hence, based on the research gaps and theoretical foundation, this study aimed to explore the possible mediation relationships between depression, anxiety, emotional competence, and COVID-19 exposure, and understand the long-term developmental trend of mental health status among Chinese adolescents through a longitudinal design study, before and during COVID-19. This longitudinal analysis involved six major variables including T1 depression, T2 depression, T1 anxiety T2 anxiety, T2 emotional competence, and T2 COVID-19 exposure. As previous research suggested that people with different mental disorders (e.g., depression and anxiety) before COVID-19 could display dissimilar developing trends in psychological status during COVID-19 (15). It is imperative to examine the mental health symptoms (i.e., T1 depression and T1 anxiety) before COVID-19 and accurately detect changes in mental health via a longitudinal study during the pandemic.

## Materials and Methods

### Dataset

A face-to-face interview questionnaire was used to collect data from students in two middle schools and three high schools. Data were collected from two-wave studies named Chengdu Positive Child Development (CPCD) survey (8). Time 1 (T1) data were collected between December 23, 2019, and January 13, 2020, before the outbreak of COVID-19 in China. Time 2 (T2) data were collected between June 16, 2020, and July 8, 2020, when the epidemic was under control in China and schools were re-opened. All participants were informed of the research purpose, privacy measures, and data retained in the signed consent form. This study was approved by the Research Ethics Committee of the University. Questionnaires were distributed to 10,370 participants. A total of 8,749 valid questionnaires were returned in the T1 study (51.62% males, M_age_ = 12.02, SD = 2.30, response rate = 84.37%). A total of 7,958 participants completed the second-wave T2 (51.67% males, M_age_ = 11.74, SD = 2.15, response rate = 76.74%). A total of 791 participants were lost to follow-up at T2. This study only included a sample of 7,958 participants who completed the two-wave study.

### Measures

#### Depression

The past-week symptoms of depression were measured using the Chinese version of the Center for Epidemiologic Studies Depression Scale (CES-D) in both wave studies (32). The depression scale consists of 20 items, each rated on a four-point scale from 0 (not at all) to 3 (a lot). An example item is, “I felt lonely, and I don't have a lot of friends.” Higher scores indicated a higher severity of depressive symptoms and total scores over 15 can indicate significant levels of depressive symptoms. The Chinese version of the CES-D has good validity and reliability in Chinese samples (32, 33). In this study, the scale showed good internal consistency (Cronbach's alphas were > 0.87 in both wave data).

#### Anxiety

The last 3-month symptoms of anxiety were assessed using a subscale (Generalized Anxiety Disorder) of the Chinese Screen for Child Anxiety Related Emotional Disorders (SCARED) in each wave (34). The subscale contains nine items in total. Participants rated each item on a three-point scale from 0 (never) to 2 (often). A sample item is, “I worry about whether other people like me.” Higher scores indicated a greater level of anxiety. Total scores over 9 can indicate significant levels of anxiety symptoms. The Chinese version of the SCARED has been shown to have good validity and reliability in previous studies (34, 35). In the current study, the internal consistency for this measure was good (Cronbach's alphas were >0.86 in both wave data).

#### Emotional Competence

Emotional competence was measured using a subscale of the Chinese Positive Youth Development Scale (CPYDS) in the second wave of the study (36). The CPYDS included 90 items, in which a six-item subscale was used to examine emotional competence. Each item is rated on a six-point scale ranging from 1 (strongly disagree) to 6 (strongly agree). An example item is “When I am unhappy, I can appropriately show my emotions.” The reliability and validity of the subscale were established in previous studies (36, 37). In this study, the subscale showed good internal consistency (Cronbach's alpha was 0.86).

#### COVID-19 Exposure

Based on the context of the participant population and previous studies (38, 39), nine items were developed to test the COVID-19 exposure in the second wave of the study. Four items with a four-point scale (1 = not at all; 4 = extremely severe/dangerous/possible/ability) examined the perceived severity, danger, infection risk, and prevention ability for COVID-19. One dichotomous question tested whether the participant's family had been infected with COVID-19 (1 = no; 2 = yes). Additionally, four questions with a four-point scale (1 = not at all; 4 = extreme influence) assessed the effects of diet, study, social life, and recreational activities. The total score was the sum of all items, with a higher score indicating a higher level of COVID-19 exposure.

### Statistical Analysis

There were four aspects in the data analysis using SPSS Version 24.0 (40) and AMOS Version 23.0 (41). First, descriptive analyses and correlations were conducted for all the variables. Second, the independent samples *t*-test was used to examine the differences between 7,958 participants who completed the two-wave study and 791 respondents who were lost to follow-up in the T2 study among all variables. Third, a paired-samples *t*-test was used to test the differences in levels of depression and anxiety between the first and second waves of the study. Finally, structural equation modeling was conducted to test the longitudinal mediation relationships between depression, anxiety, and emotional competence. Six potential mediation pathways were tested. Four variables—depression, anxiety, emotional competence, and COVID-19 exposure were modeled as latent variables in the current study. In addition, three covariates, including age, gender, and ethnicity, were added to examine the mediation model since they could be correlated with depression, anxiety, and emotional competence. To test the hypothesized mediation effect for statistical significance in AMOS, bootstrapping was used via 5,000 bootstrapped replications. Parameters were examined through a maximum likelihood estimation. According to prior research (42), χ^2^ statistics are usually applied for testing model fit, but they could be largely influenced by the sample size. Thus, other model fit indices were recommended to further assess the goodness fit of the model via several indexes (43), including χ^2^/df, root mean square error of approximation (RMSEA), standardized root mean square residual (SRMR), Tucker-Lewis Index (TLI), and comparative fit index (CFI). According to the recommendation of the acceptable model index, the ratio of χ^2^ to the degree of freedom should be <5.0, TLI and CFI should be higher than 0.95, and SRMR and RMSEA should be smaller than 0.05 (42).

## Results

### Descriptive Findings

The descriptive statistics, including the means, standard deviations, and intercorrelations among study variables, are displayed in Tables 1, 2. There were significant correlations between depression, anxiety, emotional competence, and COVID-19 exposure across the study waves. All scale scores showed a significantly moderate or high intercorrelation, excluding the COVID-19 exposure at T2, which was marginally correlated with emotional competence at T2 (*r* = −0.089, *p* < 0.001) after adjusting for age, gender, and ethnicity. The prevalence of depression decreased from 38.68% (*n* = 3,078) in T1 to 36.74% (*n* = 2,924) in T2. Similarly, the prevalence of anxiety significantly decreased from 13.02% (*n* = 1,036) in T1 to 12.77% (*n* = 1,016) in T2 (*p* < 0.001). Additionally, no significant differences, except for age and anxiety, were found between the study population (*n* = 7,958) and the participants lost to follow-up, based on all variables (*p*_*s*_ > 0.001).

**Table 1:** Demographics (*n* = 7,958).

**Variable**	**(*N*, %)**
**Age (7–17)**	M = 11.74, SD = 2.15
**Gender**
Male	4,112 (51.67%)
Female	3,846 (48.33%)
**Ethnicity**
Han Chinese	7,893 (99.2%)
Other Ethnicities	65 (0.8%)
**T2 COVID-19 Exposure**	M = 21.00, SD = 4.05
**T2 Emotional Competence**	M = 28.38, SD = 6.67
**Depression**
T1	M = 14.40, SD = 10.16, N_Yes_ = 3,078 (38.68%)
T2	M = 14.36, SD = 10.62 N_Yes_ = 2,924 (36.74%)
Anxiety^*^
T1	M = 3.71, SD = 3.96 N_Yes_ = 1,036 (13.02%)
T2	M = 3.33, SD = 4.07 N_Yes_ = 1,016 (12.77%)

*M, Mean; SD, Standard Deviation; N_yes_, number of participants with anxiety or depression*;

* * p <001 (Paired-sample t-test); T1, Time 1; T2, Time 2*.

**Table 2:** Means, standard deviations, and correlations among study variables (*n* = 7,958).

**#**	**Variables**	**M ± SD**	**1**	**2**	**3**	**4**	**5**	**6**
1	T1 Depression	14.40 ± 10.15	–	0.613^*^	0.530^*^	0.412^*^	−0.350^*^	0.135^*^
2	T1 Anxiety	3.71 ± 3.96	0.607^*^	–	0.424^*^	0.504^*^	−0.298^*^	0.138^*^
3	T2 Depression	14.36 ± 10.62	0.528^*^	0.430^*^	–	0.634^*^	−0.448^*^	0.183^*^
4	T2 Anxiety	3.33 ± 4.07	0.405^*^	0.518^*^	0.635^*^	–	−0.361^*^	0.187^*^
5	T2 EC	28.38 ± 6.67	−0.348^*^	−0.303^*^	−0.451^*^	−0.366^*^	–	−0.089^*^
6	T2 COVID-19	21.00 ± 4.05	0.134^*^	0.132^*^	0.180^*^	0.177^*^	−0.088^*^	–

*Left/bottom triangle is the Pearson's correlations of all study variables, right/top triangle is the partial correlation of all the variables adjusted by age, gender, and ethnicity; M, Mean; SD, Standard Deviation; EC, Emotional Competence; COVID-19, COVID-19 Exposure*;

* *p <0.001*.

### Longitudinal Mediation Model

**Figure 1:**
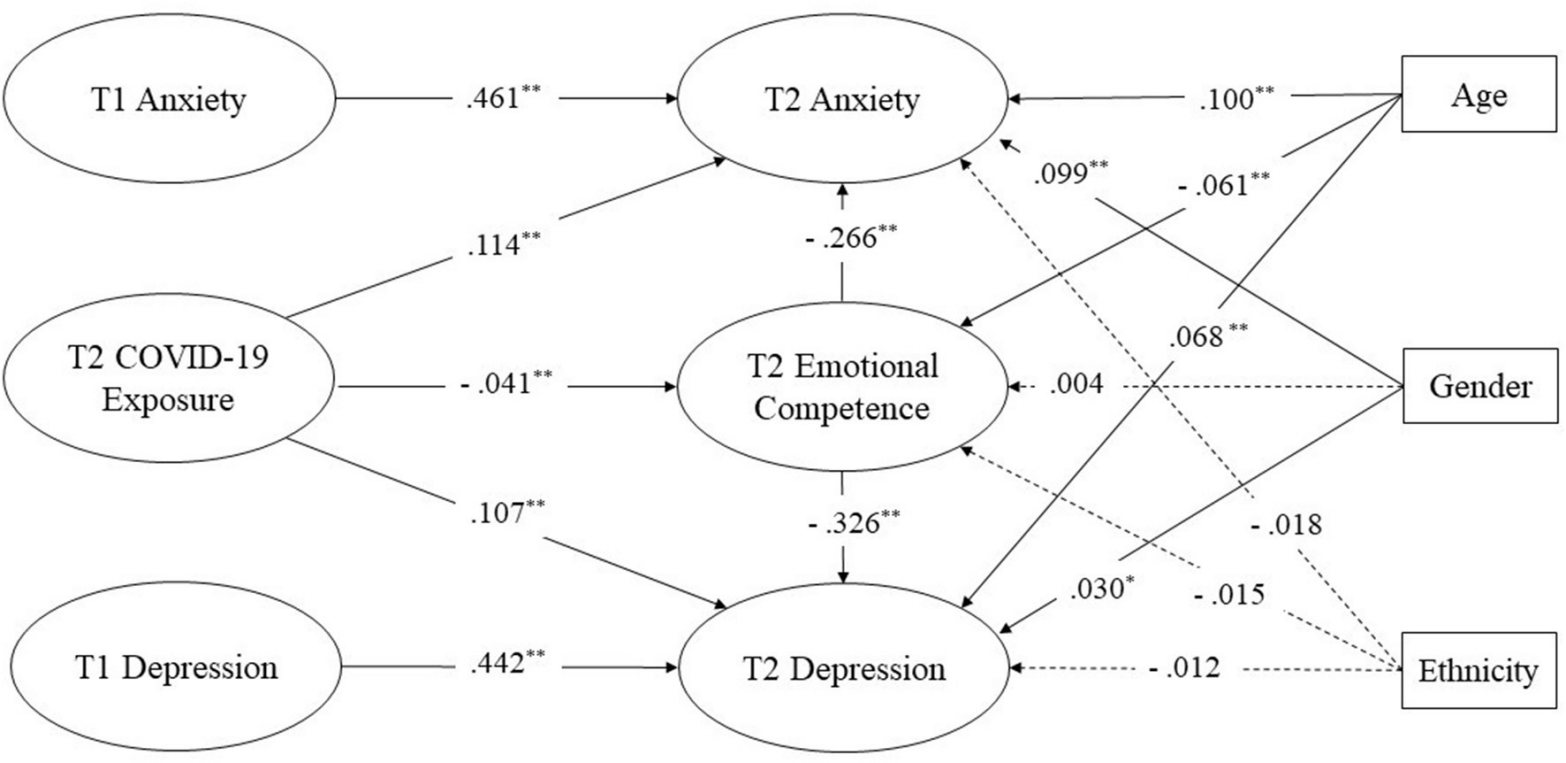
Longitudinal mediation model with standardized path coefficients. *p < 0.05, **p < 0.001; For simplicity, factor loadings of all items are not displayed in this figure. Dotted lines displayed the non-significant paths; Solid lines displayed the significant paths.

The results showed the mediation model without three covariates (i.e., age, gender, and ethnicity) and had excellent model fit (χ^2^/df = 4.029, CFI = 0.984, TLI = 0.982, SRMR= 0.037, RMSEA [90% CI] = 0.020 [0.019–0.020]). The model fit remained excellent after including covariates, age, gender, and ethnicity status (χ^2^/df = 4.974, CFI = 0.976, TLI = 0.973, SRMR = 0.038, RMSEA [90% CI] = 0.022 [0.022–0.023]) (see Figure 1). Two relationships were significantly mediated by T2 emotional competence. First, T2 emotional competence significantly mediated the relationship between T2 COVID-19 exposure and T2 anxiety (indirect effect [95% CI] = 0.011 [0.004–0.019], *p* < 0.05). Second, T2 emotional competence significantly mediated the relationship between T2 COVID-19 exposure and T2 depression (indirect effect [95% CI] = 0.013 [0.005–0.022], *p* < 0.05).

The longitudinal mediation model estimated the association between COVID-19 exposure, emotional competence, anxiety, and depression across the two waves, in which age, gender, and ethnicity were covariates. The path coefficients of the model are displayed in Figure 1, which indicates several significant direct paths, including autoregressive paths from T1 anxiety to T2 anxiety (β = 0.461, SE = 0.016, *p* < 0.001) and T1 depression to T2 depression (β = 0.442, SE = 0.014, *p* < 0.001), and direct effects from T2 COVID-19 exposure to T2 anxiety (β = 0.114, SE = 0.006, *p* < 0.001), T2 COVID-19 exposure to T2 depression (β = 0.107, SE = 0.031, *p* < 0.001), T2 COVID-19 exposure to T2 emotional competence (β = −0.041, SE = 0.015, *p* < 0.001), T2 emotional competence to T2 anxiety (β = −0.266, SE = 0.005, *p* < 0.001), and T2 emotional competence to T2 depression (β = −0.326, SE = 0.029, *p* < 0.001).

Age was significantly associated with T2 anxiety (β = −0.100, SE = 0.002, *p* < 0.001), T2 emotional competence (β = −0.061, SE = 0.005, *p* < 0.001), and T2 depression (β = 0.068, SE = 0.011, *p* < 0.001). Gender was significantly associated with T2 anxiety (β = 0.099, SE = 0.009, *p* < 0.001) and T2 Depression (β = 0.030, SE = 0.047, *p* < 0.05). However, four non-significant paths were found in this study. Ethnicity did not significantly associate with T2 anxiety (β = −0.018, SE = 0.047, *p* = 0.080), T2 emotional competence (β = −0.015, SE = 0.125, *p* = 0.169), and T2 depression (β = −0.012, SE = 0.263, *p* = 0.218), respectively. Gender was not a significant predictor of T2 emotional competence (β = 0.004, SE = 0.023, *p* = 0.700).

## Discussion

This longitudinal study explored the associations between depression, anxiety, emotional competence, and COVID-19 exposure among Chinese adolescents before and during COVID-19. To our knowledge, this is the first longitudinal study to highlight the vital mediating role of emotional competence as a possible mechanism underlying the association between COVID-19 exposure and long-term mental disorders of depression and anxiety. Additionally, the results further evidenced the negative influence of COVID-19 exposure on long-term mental health among Chinese adolescents. The results may provide a better understanding of psychological disorder development trends related to public health emergencies, highlighting the role of emotional competence as a mediator. This could benefit the development of more emotional competence interventions to improve future mental well-being among adolescents exposed to public health emergencies.

Based on the hypothesized mediation model, one of the major findings was that 6-month follow-up levels of emotional competence played a vital mediating role in the association between 6-month follow-up depression and COVID-19 exposure after controlling for gender, age, and ethnicity. Most prior studies on mental health in the COVID-19 preferred to explore its correlation between outcome variables without including potential mediators of psychological disorders (44, 45). The results from the current study suggested that emotional ability related variables, particularly the ones examining the levels of emotional skills or capabilities, could be included when exploring long-term psychological disorders in the context of the COVID-19 pandemic. It is imperative for researchers to consider variables related to emotional ability in studies on adolescents. This is because when young people are better able to manage their emotions, they are more likely to seek mental support from professionals and are more likely to recover from mental disorders (46). Other vital elements of emotional ability include the knowledge to identify mental health problems, the capacity to seek help, and the capability to recognize mental health issues (26). These aspects could be included in future studies to examine their effect on relieving long-term mental disorders among adolescents exposed to public emergencies. Moreover, this current study displayed a significantly negative association between depression and emotional competence at the 6-month follow-up, which suggested that adolescents with a higher emotional competence are likely to have lower levels of depression. This is consistent with previous research which showed that people with a better ability to recognize mental disorders are less likely to suffer from mental disorders when exposed to the COVID-19 pandemic (47). The results further emphasized the importance of improving emotional competence, such as improving Chinese adolescents' ability to recognize and modify their negative emotions.

This study found at the 6-month follow-up, that levels of emotional competence significantly mediated the association between anxiety and COVID-19 exposure. These results are consistent with the study hypotheses and supported by the transdiagnostic emotion dysregulation model which states that increasing emotional competence influences the association between early indication of psychological distress (e.g., depression and anxiety) and relieving psychological distress later (27, 48–50). This pattern also matches the transdiagnostic models of psychopathology, which claim that better emotional trends and abilities help diminish mental disorders (29, 30). For individuals with early mental health problems, future well-being can be safeguarded by improving their emotional competence (22). Additionally, there was a significant negative association at the 6-month follow-up between emotional competence and COVID-19 exposure. A meta-analysis with 31 cross-sectional studies reported a slightly higher overall prevalence of anxiety (24%) than depression (22%) among adolescents exposed to COVID-19 pandemic (12). These results suggested that promoting emotional competence could be an effective way to help adolescents with anxiety symptoms when suffering from public emergencies.

Unexpectedly, this study found that the prevalence of depression and anxiety decreased among Chinese adolescents after exposure to the COVID-19 pandemic. This result is different from the findings of most previous studies that reported the increasing of psychological disorders during COVID-19 pandemic (13, 14). One possible explanation is that adolescents with severe mental health problems were able to seek more emotional support from families or friends, increasing their emotional ability, which resulted in a decrease in the occurrence or severity of future mental health problems (51). Moreover, previous research suggested that people could receive more emotional support from society under triggering events, such as COVID-19 pandemic, than during routine times (52). When COVID-19 pandemic occurred, Chinese society provided considerable mental health support for exposed individuals, which could promote emotional competence and relieve mental disorders (53). This study also demonstrated an increase in emotional competence among Chinese adolescents after exposure to COVID-19 pandemic, which possibly resulted from the improvement in social and mental health support resources during COVID-19 pandemic in China (53). Prior research has shown that long-term mental health problems can be decreased when more emotional recognition is received (23).

This result indicating decreased mental disorders aligned with the results of smaller research among Chinese and American populations (16, 17). There are some possible explanations for this result. For example, the level of depression and anxiety decreased in Chinese adolescents because of decreased academic pressure (54). A major reason for depression and anxiety among Chinese adolescents is their interpersonal relationships (55). Many Chinese adolescents had to study at home because of COVID-19 pandemic, and they could avoid academic stress and difficult interpersonal relationships at school. Therefore, the prevalence of mental disorders could be reduced during the COVID-19 period compared with the pre-COVID-19 period (16). Moreover, a previous study suggested that COVID-19 stay-at-home regulations could provide a protective effect and context for the mental health of youth (17). Thus, there was a decreasing trend of depression and anxiety among Chinese adolescents because they could have gained more mental health support from their families during COVID-19 than pre-COVID-19 pandemic, because they were likely to have had more time with their family members at home (56).

The results suggested that ethnicity was not a significant predictor of depression, anxiety, and emotional competence in the 6-month follow-up, which was inconsistent with previous research which suggested that being an ethnicity could influence mental health and emotional competence (57, 58). One possible explanation for these results was that the differences between the other minorities and the Hans (viz., The ethic Han Chinese accounts for more than 90% of the total population in China) are narrowing because of the educational improvement, economic development, and acculturation within the Chinese population (59), which suggests that ethnic differences did not influence the mental status and emotional competence education. Furthermore, the percentage of ethnic minority participants was low in this study, compared with the Han participants, which possibly resulted in underestimating the influence of minorities on outcome variables in the structural equation model (60). Further research is suggested to balance the percentage of the minorities and the Han participants when exploring related research topics.

Based on a large sample of Chinese adolescents, the current study applied well-validated measures to explore the associations between emotional competence, depression, anxiety, and COVID-19 exposure via a complete longitudinal mediation design. The results underscored the vital role of emotional competence in influencing long-term mental disorders, such as depression and anxiety, among Chinese adolescents exposed to COVID-19 pandemic. Despite these merits, this study has several limitations that warrant discussion. First, this study utilized face-to-face interview questionnaires to collect data, which could result in response bias because of the different qualities of the interviewer (61). Future studies should apply multiple approaches to collect data, such as mixed-method research. Second, this study was based on a two-wave study to explore the mediating effect of emotional competence on the association between COVID-19 exposure and mental disorders. There are other possible factors influencing long-term mental disorders among Chinese adolescents exposed to COVID-19 pandemic, such as social support (10), psychological help-seeking (62), social media usage (63), and resilience (64). Future research is suggested to explore more possible factors relieving long-term mental disorders among adolescents exposed to COVID-19 pandemic. Finally, the mediation model could not prove the causal relationships between the variables without using the experimental method. A future study is recommended to explore the related intervention of emotional competence and its effect on long-term mental disorders among adolescents based on an experimental design (65).

## Conclusions

This study emphasized emotional competence as an effective alleviative variable against long-term depression and anxiety among Chinese adolescents exposed to COVID-19 pandemic, the negative association between psychological distress (i.e., depression and anxiety) and emotional competence, and the long-term influence of improving emotional competence on mental health when exposed to COVID-19 pandemic. Additionally, the study found a decreased trend of mental disorders, including depression and anxiety among Chinese adolescents before and during the COVID-19 pandemic, which suggests that more variables, such as family support (51), receiving social information (63), and quarantine policies (10), need to be explored for releasing the mental burden among the population exposed to COVID-19 pandemic. Concerning psychological practice, this study suggested that more government- or school-based online lectures, training, or guidebooks about improving emotional competence should be provided for adolescents exposed to the COVID-19 pandemic, which could protect exposed individuals from exacerbating mental disorders. Moreover, intervention programs targeted at reinforcing emotional competence during or after COVID-19 pandemic are necessary, which could be beneficial for strengthening emotional recognition and resilience and assisting in mental health and adaptability in future public health emergency events. For example, Kumschick and his colleagues (66) designed a literature-based intervention, named *READING AND FEELING*, to increase emotional competence. The results demonstrated the effectiveness of the *READING AND FEELING program* in improving emotional ability, based on emotional words, specific emotional literacy, and recognition of disguised moods among the youth. Furthermore, another intervention, *Health Promoting Schools Up*, was developed to effectively boost mental health by improving social and emotional competence among schoolchildren (67). Many researchers have recommended that various health support materials be provided for individuals exposed to COVID-19 pandemic to potentially relieve their long-term mental health disorders that develop due to this pandemic. These include online peer-support interventions (68), cyber-counseling (69), and digital mental health services (70), which could help to detect early psychological problems and avoid mental symptom deterioration (71–74).

## Data Availability Statement

The raw data supporting the conclusions of this article will be made available by the authors, without undue reservation.

## Ethics Statement

The studies involving human participants were reviewed and approved by Research Ethics Committee of the University. Written informed consent to participate in this study was provided by the participants' legal guardian/next of kin.

## Author Contributions

PJ and LZ did study design and data collection. WS and GY analyzed data, drafted, and submitted this manuscript together. PJ, LZ, BH, YS, and XL revised the manuscript. All authors contributed to manuscript checking and approval the final manuscript.

## Funding

This work was supported by the Fundamental Research Funds for the Central Universities (20827044B4020) and the International Institute of Spatial Lifecourse Epidemiology (ISLE).

## Conflict of Interest

The authors declare that the research was conducted in the absence of any commercial or financial relationships that could be construed as a potential conflict of interest.

## Publisher's Note

All claims expressed in this article are solely those of the authors and do not necessarily represent those of their affiliated organizations, or those of the publisher, the editors and the reviewers. Any product that may be evaluated in this article, or claim that may be made by its manufacturer, is not guaranteed or endorsed by the publisher.
